# Esophageal Cancer: Should Gender Be Considered as an Influential Factor for Patient Safety in Drug Treatment?

**DOI:** 10.1155/2019/6340567

**Published:** 2019-05-23

**Authors:** Fengxia Liu, Helin Feng, Sumin Guo, Yuhan Chen, Qingyi Liu, Feng Wu, Weikuan Gu, Baoen Shan

**Affiliations:** ^1^The Fourth Hospital, Hebei Medical University, 12 Jiankang Road, Shijiazhuang, Hebei Province 050011, China; ^2^Department of Orthopedic Surgery and BME-Campbell Clinic, University of Tennessee Health Science Center, Memphis, TN 38163, USA; ^3^Department of Oncology, Chest Hospital of Hebei Province, Shijiazhuang, Hebei 050041, China; ^4^Guang'anmen Hospital, China Academy of Chinese Medical Sciences, Beijing 100053, China; ^5^The 2nd Affiliated Hospital of Harbin Medical University, No. 7 building, Baojian Road, Harbin, Heilongjiang 161005, China; ^6^Research Service, Veterans Affairs Medical Center, 1030 Jefferson Avenue, Memphis, TN 38104, USA

## Abstract

**Aim:**

Analyze the gender difference of esophageal cancer patients in response to drug treatment.

**Methods:**

All publications on clinical trials were collected from PubMed, Scopus, and PMC. Each publication was examined to determine whether the publication is a clinical trial and whether data on gender difference were reported.

**Results:**

Selected from a total of 191 publications, data from 7 trials with a total of 2041 patients were evaluated for gender differences. These clinical trials involve different drugs and disease phenotype. A significant difference was obtained between male and female groups from Student's t-test. There is no conclusive result on age, ethnicity, tumor size, and drug influence.

**Conclusions:**

Gender difference in response to treatment potentially most likely exists in esophageal cancer patients, regardless of age, race, and drugs.

## 1. Introduction

The National Institutes of Health (NIH) recognized in 2014 that sex and gender play a role in how health and disease processes differ across individuals and requested that sex be factored into research designs, analyses, and reporting in vertebrate animal and human studies as a biological variable [1]. Esophageal cancers, including cancer arising from the gastroesophageal junction, are challenging diseases worldwide. Surgery has been the only option for treatment for the past few decades. Recently, positive results from several new drugs, including ramucirumab, everolimus, capecitabine, and oxaliplatin, raise the hope for chemotherapy [2–10].

The gender difference in esophageal cancer incidence has been well known [8, 9]. Particularly, the male to female ratio of esophageal cancer incidence is 3:1. Gender differences in medicine have commanded attention in recent years [11–15]. Health disparity research is one recently emphasized program at the US National Institutes of Health [1]. Potential sex differences in genes in the epidermal growth factor receptor (EGFR) axis have been reported in humans and in rat models [16–20].

In this review, safety data on patients in phase III trials over the past ten years were examined. The data on hazard ratio (HR) were analyzed for the gender, age, and gender and drug interactions. The emphasis is on whether there are differences between and among gender, age, and drugs in these clinical trials.

## 2. Methods

All of the possible publications on clinical trials were collected from PubMed, Scopus, and PMC. Each publication was examined to determine whether the publication is a clinical trial and whether data on gender, age, and drugs were reported in the clinical trials. A minimum of 100 patients in the clinical trial was also used as selection criteria. This selection criterion is designed to avoid potential bias because of the small number of female patients in sparsely populated trials. The female patients in the reported clinical trials are usually within the range between 10% and 25% of total participants [2, 4, 5, 7–10]. The number of female patients in a clinical trial with less than 100 patients would be too small to be used in the analysis of sex differences. For all three databases, searches were limited to the title of the article in English and those articles published between 2005 and 2016, inclusive.

**Figure 1:**
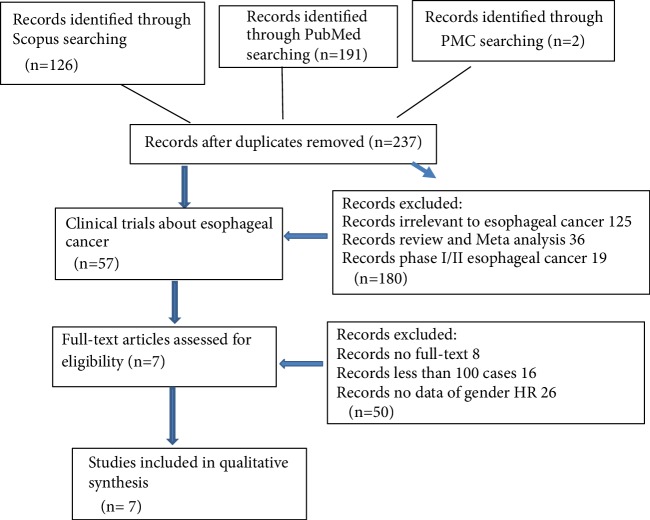
Flow diagram of selecting studies.

For searching PubMed, key words “esophageal cancer trial phase-III” were used to retrieve the publications. In total, 237 articles were identified from the three databases after duplicates were removed (Figure 1). After the titles and abstracts of these studies were examined, 180 papers were excluded either because they were not trials, not associated with esophageal cancer, or because they were phase I/II trials. Another 50 trials were excluded due to the following reasons: full-text was not available for 7 studies; 14 studies had fewer than 100 cases; and 29 studies did not present gender HR data. Ultimately, seven trials were included for systematic review [2, 4, 5, 7–10], all found in PubMed.

For Scopus, a total of 126 publications were identified. They were all excluded for the following reasons: 79 were duplicated with PubMed, 10 were reviews, 4 were phase I/II trials, 3 cases had fewer than 100 patients, and 2 cases had more than 100 patients, but without gender HR information.

For PMC, 2 cases were identified but were duplicated in PubMed.

Altogether, data from seven publications reporting results of clinical trials on the treatment of esophageal cancer were used for our analysis.

## 3. Results

### 3.1. Sex Balance in the Study of Clinical Trials of Esophageal Cancer Has Not Been Adequately Addressed

Our literature review indicated that the majority of the studies did not analyze female and male patients separately. For example, from PubMed, among 30 clinical trials that had more than 100 participants in the trials, 23 of them did not analyze gender influence on effectiveness of the drugs. Only in seven trials was the gender difference analyzed. The ratio of gender-analyzed and nonanalyzed trials is 1:4. All of these 23 nonanalyzed trials were reported after 2010, when the public had begun to demand attention to sex differences [13, 14]. In particular, 13 were published on or after 2014, when the NIH imposed the policy on gender analysis for its supported studies [1]. Accordingly, the countries of origin for these 13 publications were examined. Based on the institute addresses of the first authors, only one of these 13 first authors was from the US, but possibly reporting an international collaborative clinical trial because coauthors were from large number of countries such as China, Korea, Poland, Ukraine, Brazil, Italy, Chile, Russia, Belgium, and France [20]. The rest of the trials were conducted from countries in Europe and Asia. These 13 clinical trials included 3014 patients, which would be a tremendous resource for the analysis of gender differences if such analysis was conducted as part of the trials' reporting.

### 3.2. Gender Effect on the Efficacy of Drugs

We are able to obtain separated hazard ratio data of female and male subjects from 7 studies. These 7 trials have a total of 2041 patients (Table 1), including 326 female patients. Among the 7 publications, one reported by Crehange and colleagues [10] analyzed both progression-free survival (PFS) and overall survival (OS) ratio (Table 1). In both cases, female patients showed a better response to chemoradiotherapy followed by surgery [10]. Two studies provided PFS [2, 7] only, while one provided OS only [8]. One compared the death rate between sexes [5]. One study calculated the PFS for both metastatic recurrence and locoregional recurrence [4]. Within these 7 studies, the hazard ratios of men were all higher than that of women except for one trial, which studied the comparison of preoperative chemotherapy or preoperative C/RT groups [8]; this study had the smallest patient population among the 7 studies. We first conducted a blunted Student's t-test. For this test, we listed every hazard ratio of men and women, without separating the nature of the ratio. The average HRs in female and male patients were 0.811 and 1.07, respectively. In the t-test, the data were treated as paired and analysis was conducted as a two-tailed test. A P value of 0.030 was obtained from our t-test. Because one study provided data of both PFS and OS, we analyzed the data from this trial separately with either PFS or OS from this study [10]. The P values for inclusion of only PFS or only OS from this study [10] were 0.037 and 0.072, respectively.

Thus, it is most likely that there are gender disparities existing for patients as to the HR in drug treatments for patients with esophageal cancer.

### 3.3. Age Effect on the Efficacy of Drugs

Five of these clinical trials provided data on the HR values of age differences (Table 2) [4, 5, 8–10]. However, there was no evidence showing age differences in the drug treatment of esophageal cancer. From the two studies that compared age difference, the ratio is 1.0 and 1.01 (Table 1) [8, 9]. The other three studies made comparisons between age 60 and above. In the comparison of chemoradiotherapy and surgery versus surgery alone, authors obtained a ratio of 1·49 and 0.88 in comparing patients of age >60 versus. <=60 for locoregional recurrence and univariable HR, respectively [4]. In the other study [10], the PFS and the OS patients of age >60 versus <=60 were 1.43 and 1.53, respectively. In another study, patients were divided into three age groups, <60, 60-69, and >=70 [5]. The death risks were 0.85, 0.92, and 0.93, respectively.

Thus, a definitive conclusion cannot be drawn from the inconsistencies of age effects from these studies. In order to better understand the age effect, a consensus on the age grouping among researchers may be necessary. At present, we do not see any age effect on the HR values of gender differences among these patients.

### 3.4. Gender and Age Interaction

At present, there is no separate statistical data on the age of women and men. Although it is known that esophageal cancer is 4 times more common among men than among women, there is no report on the age difference between women and men. Since in our selected trials there is no difference between age groups, age is ruled out as a potential factor that influences gender differences.

### 3.5. Ethnic Groups and Gender Difference

Among these seven studies, one was from an Asian group [5]. Although the risk of death rate in female patients is less than that in male patients, statistically there is no significant difference between these two groups.

Among the rest of the six trials, four are from European countries, one from North America 8 and one from Australia [10]. Within these studies, no ethnic groups were revealed or analyzed from the patients. We assume that the results in these trials were mainly from North American and European populations. Based on these studies, the average HR values are 0.7075 and 0.9538 for women and men, respectively. Student's t-test for the HR between sex groups is 0.0345. These can only be interpreted as evidence that these two groups are likely significantly different, because one of these trials provided both progression-free survival (PFS) and overall survival (OS) ratios while others provided only one of these ratios. In one of our tests, OS data from this study were deleted. A P value of 0.0416 was obtained, and the average HR value of women was 0.5475 while that of men was 0.7878. We next eliminated PFS data from this study. A P value of 0.0852 was obtained, and the average HR values were 0.655 and 0.8375 for women and men, respectively.

Overall, gender differences exist in North American and European populations. Moreover, considering the fact that most European or North American and Australian populations are mixtures of ethnically diverse groups, gender differences may potentially exist in all ethnic groups.

### 3.6. Effect of Pathological Characteristics

The effect on treatment of pathological characteristics were examined (Table 3) for any significant influence. These seven studies used a variety of methodologies in characterization of patients. These methods included WHO performance status, differentiation status, length of tumor, histology/squamous cell carcinoma, and location of tumor. Because of the different methodologies in different studies, a comprehensive statistical comparison is difficult. Nevertheless, a tentative conclusion was obtained through examination of these data. In WHO performance status, ≥1 versus 0 seemingly shows some difference, with a P value of 0.0513. While the average HR values of 0 grade are 0.873, the HR for 1 grade is 1.01. There were not enough data to analyze the influence of tumor differential and length of tumor on drug treatment. In the comparison between squamous cell carcinoma (SCC) and other types, current data did not produce a significant P value, the P value beings 0.179. Interestingly, there is no difference between the location of the cancer, comparing the upper and lower part of the esophagus (P=0.398). Overall, it is not likely that pathological characteristics influence gender differences in treatment.

### 3.7. Drug and Gender Difference

We next examined whether particular drugs are linked to gender differences. Among all these trials, only one did not show gender difference 8. That study is the comparison of preoperative chemoradiotherapy (C/RT) versus surgery alone. The two drugs used in this study, cisplatin and paclitaxel, were the same as in the other studies. For example, a study by Oppedijk et al. [7] analyzed recurrence patterns in patients treated with either surgery alone or surgery plus preoperative chemoradiotherapy (CRT), which consisted of five weekly courses of paclitaxel and carboplatin combined with concurrent radiation. In the study by Swisher et al. [8], treatment consisted of three cycles of cisplatin and 5-fluorouracil (5-FU) before surgery. Two courses of treatment with cisplatin and 5-FU were conducted in the study by Crehange et al. [9]. In the study by Burmeister et al. [10], randomly assigned treatments with cisplatin and 5-FU were also conducted. Therefore, drugs were not the cause that led to the different results from this study. The small number of total patients, a total of 157, and the small number of female patients, only 16 in total, were considered to be the reasons that this study did not show gender differences. Thus, drugs used in these clinical trials did not influence the gender difference.

### 3.8. Conclusions and Prospective

The collective analysis of previous studies suggests that gender differences in response to drug treatment in esophageal cancer is potentially all across the spectrum, regardless of age, race, and drugs. A P value from t-test with combined PFS and OS values from seven qualified studies reached a significant level at 0.030. Separated analysis of PFS and OS values produced P values of 0.037 and 0.072. Unlike previously suggested [21], our analysis indicated that gender difference seems to not be affected by other factors. Because no age effect was found on drug treatments, age may not be one of the factors that influence gender difference. Also, data showed no evidence on the gender effect from different drugs. Gender differences were found from North American and European populations with P value of 0.0345. The gender differences in the Asian population need to be confirmed with more studies. Therefore, gender differences in the response to treatment of esophageal cancer should demand attention from basic researchers for understanding its molecular mechanism, as well as from clinicians for potential treatments based on gender such as dosage or frequency of drugs.

This review focuses on a very critical issue, the hazard ratio in clinical trials, and conducted a comprehensive analysis on the available data. We included data from all the major clinical trials in the past ten years, which represent the general picture in drug development for gastric cancer. In our initial literature searching, the excluded reports on clinical trials are those that are either did not conduct the subgroup analysis between female and male patients or were otherwise not accessible for this review. In general, these have relatively small patient populations [3, 6].

To determine the causes for different responses to drugs, such as whether the differential response is genetic, physiological, behavioral, or simply due to life habits, future studies and data collections are needed. Most likely, the interactions between genetic physiological complexes and molecular mechanisms of drugs play an important role. Subgroup analysis in future clinic trials, with either smaller or larger numbers of patients, is essential for clarifying these critical issues in drug applications.

## 4. Executive Summary

The male to female ratio of the esophageal cancer incidence is 3:1. An important question is whether there is a gender difference in response to drug treatments in patients of esophageal cancer.

Hazard ratio (HR) data were used to compare gender differences. We have analyzed results using a total of 2198 patients from 7 selected clinical trials.

Student's t-test indicated that there is a gender difference in the HR during drug treatment.

Tests indicated that age and pathology status do not influence the HR between male and female patients.

Different drugs may influence the HR between male and female patients.

### 4.1. Therapeutic Implications

The gender difference in HR suggests that physiological and biological differences in cancer development and response to drug treatments exist between female and male patients.

Such gender differences affect the efficacy of drug treatment.

Age should not be considered as a factor that influences drug treatments for esophageal cancer.

Future research and clinical trials should be designed to be sensitive to and account for possible gender differences.

## Figures and Tables

**Table 1:** Summary of hazard ratio of men and women with esophageal cancer in clinical trials.

Authors/reference #/year	N (%Men)	N (%women)	N (Women/Men)%	PFS HR (95% CI)	OS HR (95% CI)	Cox regression analysis	Death ratio HR (95% CI)
Robb W.B. et al. /[4]/2015	146/170 =85.9	24/170 =14.1	24/146 =16.4	metastatic recurrence F/M Univariable 0.26(0.06-1.09) Multivariable 0·35 (0·08, 1·47)	-	locoregional recurrence Univariable F/M 0·68 (0·24, 1·90)	-

Zhao Y, et al. /[5]/2015	297/346 =85.8	49/346 =14.2	49/297 =16.5	-	-		Death M: 0.89(0.75-1.07) F:0.83(0.57-1.21)

Dutton S.J. et al. /[2]/2014	372/449 =82.9	77/449 =17.1	77/372 =20.7	M 0.82 (0.67–1.01) F 0.70 (0.44–1.12)	-		-

Oppedijk V. et al. /[7]/2014	298/374 =80	76/374 =20	76/298 =25.5	M/F 1.12(0.67-1.87)	-		-

Swisher S.G. et al. [8]/2010	141/157 =90	16/157 =10	16/141 =11.3	-	M:1.0(0.5-1.7) F: 1.0		-

Crehange G. et al./[9]/2007	412/446 =92.4	34/446 =7.6	34/412 =8.3	M/F1.4(0.8-2.45)	-		-

Burmeister B.H. et al./[10]/2005	206/256 =80.5	50/256 =19.5	50/206 24.3	PFS M 0.93(0.67-1.29) F 0.42(0.19-0.91) M/F:1.28(0.86-1.90) OS M/F 1.36(0.93-1.99)			

**Table 2:** Summary of hazard ratio of <65 versus >=65 with esophageal cancer in clinical trials.

Authors/reference #/year	N <65	N >=65	Age mean (range)	Age Difference HR (95% CI):	PFS (> 60 versus ≤ 60 years)	Univariable HR Age (> 60 versus ≤ 60 years)
Robb W.B. et al. /[4]/2015	-	-	57·8 (36·9–76·4)		locoregional recurrence 1·49 (0·81, 2·76)	metastatic recurrence 0·88 (0·49, 1·60)

Zhao Y, et al. /[5]/2015	174	172	59 (23-90)	(<65 vs >=65) HR (95% CI):<60: 0.85(0.69-1.05) 60-69: 0.92(0.67-1.25) >70:0.93(0.62-1.39)	-	-

Dutton S.J. et al. /[2]/2014	-	-	64.8(58.0-70.7)	-	-	-

Oppedijk V. et al. /[7]/2014	-	-	60(36-79)	-	-	-

Swisher S.G. et al. [8]/2010	-	-	58(23-77)	1.0 (0.9–1.1)	-	-

Crehange G. et al./[9]/2007	-	-	59	1.01 (0.99 to 1.03) Local Relapse-Free Survival 1.01 (1.00 to 1.02) Overall Survival	-	-

Burmeister B.H. et al./[10]/2005	-	-	62(28-83)	-	PFS HR:1.43(1.06-1.99) P:0.02	Overall survival 1·53 (1·14–2·06)

**Table 3:** Summary of hazard ratio of pathological characteristics with esophageal cancer in clinical trials.

Authors/reference #/year	WHO performance status	Drug vs Preoperative chemoradiotherapy (CRT)	Differentiation status	Length of tumor	Histology/ squamous cell carcinoma (SCC)	Location of tumor
Robb W.B. et al. /[4]/2015	≥1 vs 0 locoregional recurrence HR:0.97(0.46,2.04) metastatic recurrence 0·67 (0·31, 1·43)	nCRTs vs surgery only Univariable HR:0.80(0.45-1.43) Mulivariable HR:1.03(0.56-1.93) P:0.917	-	-	Adenocarcinoma vs SCC HR:0.96(0.47-1.96)	Below vs above carina HR:1.09(0.34-3.52)

Zhao Y, et al. /[5]/2015	0 HR:0.85(0.69 -1.04) 1 HR:0.97(0.76-1.23)	Perioperative vs preoperative 5-year relapse free HR:0.62(0.49-0.73) HR for death:0.79(0.59-0.95)	-	≥8.0cm HR:0.95(0.73-1.24) <8.0cm HR; 0.86(0.71-1.04)	-	Upper/middle HR:0.86(0.66-1.13) Lower HR:0.89(0.73-1.08)

Dutton S.J. et al. /[2]/2014	0 HR:0.67(0.46-0.98) 1 HR:0.83(0.64-1.08) 2 HP:0.81(0.54-1.23)	Overall survival Gefitinib vs placebo HR:0.90	-	-	Adenocarcinoma HR:0.81(0.65-1.01) Squamous cell HR:0.72(0.48-1.08)	Oesophageal HR:0.83(0.64-0.99) Junctional I/II HR:0.73(0.48-1.09)

Oppedijk V. et al. /[7]/2014		Treatment arm(S vs S+CRT) Univariable HR:0.37(0.23-0.59) Multivariable HR:0.50(0.29-0.86)	-	≤5 cm vs >5cm HR:0.89(0.54-1.46)	SCC vs AC Univariable HR:0.70(0.44-1.12) Mulivariable HR:0.49(0.29-0.82)	-

Swisher S.G. et al. [8]/2010	1 vs 0 HR:1.24(0.91-1.70)	Preoperative C/RT vs preoperative C Overall survival HR:0.58(0.37-0.90) Disease free survival HR:0.55(0.35-0.85)	Well/moderate HR:1.0 Poor/undifferentiated HR:1.3 P:0.2 Unknown HR:1.1 P:0.7	-	-	Upper/middle HR:1.0 Lower HR:0.8(0.5-1.4)

Crehange G. et al./[9]/2007	-	P-RT vs SC-RT Univariable HR:0.87(0.68-1.11) Multivariable HR:0.83(0.63-1.08)	Dysphagia:grades 1-3 vs grades 4-5 HR:1.22(0.88-1.68)	HR:1.03(1.01-1.05) P:0.001	-	-

Burmeister B.H. et al./[10]/2005	1 vs 0 HR:1.24(0.91-1.70)	CRT+S vs S PFS HR:0.82(0.61-1.10) Overall survival HR:0.89(0.67-1.19)	Well/moderate HR:0.69(0.43-1.12) Poor HR:0.92(0.59-1.45)	>5cm HR:0.72(0.44-1.17) ≤5cm HR:0.83(0.57-1.21)	Squamous HR:0.47(0.25-0.86) Non-squamous HR:1.02(0.72-1.44)	Lower HR:0.97(0.70-1.33) Middle or upper HR:0.38(0.17-0.87)

## Abbreviations

EGFR: Epidermal growth factor receptor
HR: Hazard ratio
NIH: National Institutes of Health
OS: Overall survival
PFS: Progression-free survival
VEGFR-2: Vascular endothelial growth factor receptor-2
WHO: World Health Organization.
